# Molecular evidence of *Culex pipiens* form molestus and hybrids pipiens/molestus in Morocco, North Africa

**DOI:** 10.1186/1756-3305-5-83

**Published:** 2012-04-27

**Authors:** Fadila Amraoui, Mhamed Tijane, Mhammed Sarih, Anna-Bella Failloux

**Affiliations:** 1 Institut Pasteur du Maroc, Laboratoire des Maladies Vectorielles, 1 Place Louis Pasteur, Casablanca, 20360, Morocco; 2 Faculté des Sciences, Laboratoire de Biochimie et Immunologie, 4 avenue Ibn Battouta, Rabat, BP 1014 RP, Morocco; 3 Institut Pasteur, Department of Virology, Arboviruses and Insect Vectors, 25-28 rue du Docteur Roux, Paris, 75724, France

**Keywords:** *Culex pipiens* complex, Microsatellite, Molecular taxonomy, Morocco, North Africa

## Abstract

**Background:**

*Culex pipiens* L. is the most widespread mosquito vector in temperate regions including North Africa. *Cx. pipiens* has two recognized forms or biotypes; pipiens and molestus are morphologically indistinguishable with distinct behavior and physiology that may influence their vectorial status. In our study, we prospected for the different forms of *Cx. pipiens* in Morocco.

**Methods:**

*Cx. pipiens* larvae were collected in 9 sites throughout Morocco during summer 2010 and reared until imago stage. *Cx. pipiens* was identified using diagnostic primers designed for the flanking region of microsatellite CQ11.

**Results:**

We established the presence of both forms of *Cx. pipiens* and their hybrids in Morocco.

**Conclusions:**

Molecular identification provides the first evidence of the presence of *Cx. pipiens* form molestus in Morocco and hybrids between pipiens and molestus forms in North Africa. The epidemiological implications of our findings are discussed.

## Background

The *Culex pipiens* complex includes several species; *Cx. pipiens pipiens* Linnaeus, 1758 and *Cx. pipiens quinquefasciatus* Say, 1823 are the most ubiquitous mosquitoes in temperate and tropical regions, respectively. *Cx. p. pipiens* has two distinct forms or biotypes: form pipiens and form molestus which are morphologically indistinguishable and differ in physiology and behavior. *Cx. pipiens* form pipiens is subjected to diapause (heterodynamic), is anautogeneous (only lays eggs after a blood-meal), and eurygamous (unable to mate in confined spaces). On the other hand, *Cx. pipiens* form molestus Forskal, 1775 does not diapause (homodynamic), is autogeneous (lays first batch of eggs without taking a blood-meal) and stenogamous (mates in confined spaces) [1,2]. In addition, the biotypes molestus and pipiens occupy distinct habitats in Russia and the northeastern United States. Indeed, molestus form occurs in underground areas in urban settings while pipiens form lives aboveground [3,4]. In Europe, sympatric occurrence of both biotypes has been observed in aboveground habitats as well as in underground habitats [5-7]. The two forms did not seem to be genetically isolated and were reported to hybridize in the United States and Europe [6-8]. They have different trophic preferences: pipiens biting mainly birds and molestus feeding on mammals, whereas hybrids exhibit an opportunistic behavior and can readily feed on both hosts. These feeding patterns are thought to influence the transmission of avian and mammalian pathogens.

In North Africa, *Cx. p. pipiens* is a competent vector of several pathogens infecting animals and humans including West Nile virus [9], Rift Valley Fever virus [10-12] and filarial worms [13-16]. Based on morphological characters, behavioral and reproductive specializations, the mosquito *Cx. p. pipiens* was described in the North African region [17-25]. Nevertheless, these classical characters present limited value. Therefore, our study aims to identify members of the *Cx. pipiens* complex present in Morocco based on a molecular identification.

## Methods

**Figure 1:**
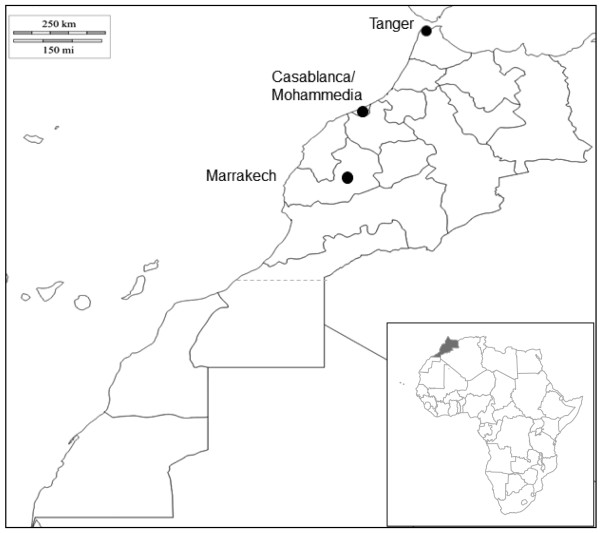
Localization of the collection sites in Morocco.

Mosquitoes were collected as larvae using the “dipping” sampling method during summer 2010 from three Moroccan regions (Figure 1). A total of 9 sites were classified according to the habitat (urban, suburban or rural) and the type of breeding site (aboveground or underground). Fourth instar larvae were used for morphological identification [26] and reared until imago stage at 28 ± 1°C with 80% relative humidity and a 16 h:8 h photoperiod. Emerged adults were conserved at −20°C for subsequent molecular characterization.

**Figure 2:**
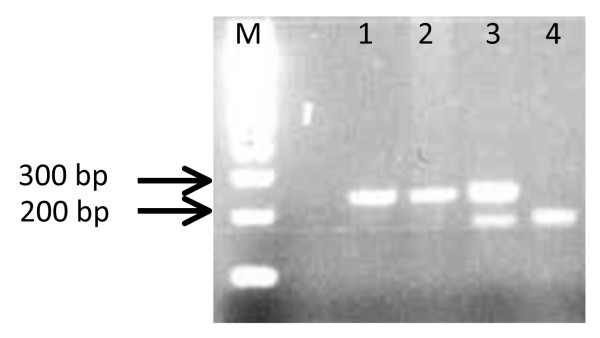
**Example of PCR amplification of the flanking region of the CQ11 microsatellite of*Culex pipiens*collected in an underground site in Casablanca (Morocco).** DNA was extracted from individual mosquitoes and identified by PCR amplification of the flanking region of the CQ11 microsatellite. Lane M: 100-bp size marker; Lane 1: control *Cx. pipiens* form molestus from Japan; Lane 2: molestus form; Lane 3: pipiens form; Lane 4: hybrid form.

DNA extraction from F0 individuals was performed using the method of DNAzol as described in the manufacturer’s protocol. Specimens were identified as belonging to the *Culex pipiens* complex using a multiplex PCR assay described in Bahnck and Fonseca (2006) [27]. The locus CQ11 was used to distinguish between the two forms of *Cx. pipiens*. The DNA fragment size amplified varied between pipiens and molestus allowing us to distinguish the two forms in a single PCR reaction (Figure 2). Specimens of *Cx. pipiens* molestus from Japan were used as control.

## Results and Discussion

A total of 214 adults were characterized by PCR and frequencies of different forms are represented in Table 1. Overall, 52.3% of adults tested were homozygous for the 200 bp fragment which is characteristic of the pipiens form, 22% were homozygous for the 250 bp fragment identifying the molestus form and the remaining (25.7%) corresponded to hybrids.

**Table 1:** **Frequency of forms of the*Culex pipiens*complex in Morocco**

City	Habitat	Breeding site	Pipiens	Molestus	Hybrids
		(Ground)	form (%)	form (%)	(%)
Tanger	Urban	Above	69.6 (16)	8.7 (2)	21.7 (5)
	Sub-urban	Above	52.2 (12)	34.8 (8)	13 (3)
	Rural	Above	62.5 (15)	8.3 (2)	29.2 (7)
Casablanca/	Urban	Above	31 (9)	17.2 (5)	51.8(15)
Mohammedia	Urban	Under	25 (8)	59.4 (19)	15.6 (5)
	Sub-urban	Above	53.6 (15)	17.8 (5)	28.6 (8)
Marrakech	Sub-urban	Above	60.9 (14)	8.7 (2)	30.4 (7)
	Rural	Above	78.3 (18)	4.3 (1)	17.4 (4)
	Rural	Under	55.6 (5)	33.3 (3)	11.1 (1)

*Cx.pipiens* larvae were collected at various sites in Morocco, reared to adults and identified by PCR amplification of the flanking region of the CQ11 microsatellite. In brackets, number of tested mosquitoes.

This study provides the first molecular evidence for the presence of *Cx. pipiens* form molestus in Morocco and hybrids in North Africa.

The molestus form has been described as a distinct species, *Cx. molestus* Forskal, 1775 from autogeneous Egyptian specimens. Because *Cx. pipiens* form molestus is stenogamous and autogenous, it colonizes underground areas in urban settings [3] with limited geographic distribution throughout the world. In our study, *Cx. pipiens* form pipiens and form molestus were found in urban, suburban and rural habitats. Indistinctly, the two forms co-occur in aboveground and underground breeding sites. Sympatric distribution of the biotypes molestus and pipiens in surface breeding sites has been observed in southern Europe and the United States [5,6,8] and in underground breeding sites in North Europe [7].

## Conclusions

Until now, hybrids were mainly reported in the United States [4,8] and South Europe [6]. Our findings corroborate the presence of hybrids in all breeding sites sampled. Hybrids between molestus and pipiens forms are considered of great epidemiological importance. They exhibit intermediate physiological and behavioral traits [28] and can readily feed on avian and mammalian hosts [8,29]. This opportunistic biting behavior will potentiate the role of *Cx. pipiens* as a bridge-vector for the transmission of pathogens such as West Nile virus, from birds (amplification hosts) to humans [8,30].

## Competing interests

The authors declare that they have no competing interests.

## Authors’contributions

FA carried out mosquito genotyping, contributed to the interpretation of results and drafted the manuscript. MT participated in the design of experiments. MS participated in the design of experiments and mosquito collections. ABF designed the experiments and drafted the manuscript. All authors read and approved the final manuscript.
